# Colombian consensus on the care of critically ill patients with suspected or confirmed severe yellow fever

**DOI:** 10.1016/j.lana.2025.101144

**Published:** 2025-06-11

**Authors:** Alex Julián Forero-Delgadillo, Jeison Andrés Morales-Olivera, Julián Fernando Celis-Guzmán, Omar Eduardo Zapata-Díaz, Gustavo Adolfo González-Varona, Camilo Alberto Acevedo-Bedoya, Rómulo Salazar-Fernández, Jaime Orlando Ordoñez, Henry Robayo-Amortegui, Alejandro Quintero-Altare, Diana Catalina Ramírez-Reyes, Juan Diego Melo-Pedraza, Juan Olivella-Gomez, Jessica María Forero, Abraham Katime-Zuñiga, Wilmer E. Villamil-Gomez, Alfonso J. Rodriguez-Morales, Ricardo Buitrago-Bernal, Fernando Bozza, Luis Felipe Reyes

**Affiliations:** a Clinical and Translational Medicine Research Group, Critical Physiology Subdivision, Intensive Care Department, Hospital Federico Lleras Acosta, Ibagué, Colombia; b Intensive Care Department, Hospital Federico Lleras Acosta, Ibagué, Colombia; c School of Medicine, Faculty of Health Sciences, Universidad del Tolima, Ibagué, Colombia; d Department of Critical Care Medicine, Extracorporeal Life Support Unit (USVEC), Fundación Clínica Shaio, Bogotá, DC, Colombia; e Critical Care Medicine, School of Medicine, Universidad de La Sabana, Chía, Colombia; f Department of Pediatric Nephrology, Funación Valle del Lili, Cali, Colombia; g Unisabana Center for Translational Science, School of Medicine, Universidad de La Sabana, Chia, Colombia; h Programa de Medicina, Universidad del Magdalena, Santa Marta, Magdalena, Colombia; i Servicio de Infectología, Universitario Julio Méndez Barreneche, Santa Marta, Magdalena, Colombia; j Comité de Medicina Tropical, Zoonosis y Medicina del Viajero, Asociación Colombiana de Infectología, Bogotá, DC, Colombia; k Centro de Investigación en Ciencias de la Vida, Universidad Simón Bolívar, Barranquilla, Colombia; l Faculty of Health Sciences, Universidad Científica del Sur, Lima, Peru; m Grupo de Investigación Biomedicina, Facultad de Medicina, Fundación Universitaria Autónoma de las Américas-Institución Universitaria Visión de las Américas, Pereira 660003, Risaralda, Colombia; n Unidad de Cuidados Intensivos, Fundación Clínica Shaio, Bogotá, DC, Colombia; o Department of Critical Care, D'Or Institute for Research and Education (IDOR), Rio de Janeiro, Brazil; p ISARIC, Pandemic Sciences Institute, University of Oxford, Oxford, UK; q National Institute of Infectious Disease Evandro Chagas (INI), Oswaldo Cruz Foundation (Fiocruz), Rio de Janeiro, Brazil

**Keywords:** Yellow fever, Consensus, Critical care, Epidemic, Colombia

## Abstract

Yellow fever is an arboviral disease transmitted by *Aedes*, *Haemagogus* and *Sabethes* mosquitoes. It features both urban and jungle transmission cycles. Its incidence has risen in Colombia due to deforestation, human expansion, and climate change. The disease can progress from a nonspecific febrile stage to a severe intoxication phase, characterised by multiple organ failure and high mortality rates. This consensus establishes criteria for early identification and management of severe yellow fever and recommendations for admission to the Intensive Care Unit in cases of liver dysfunction, kidney failure, or shock. An individualised haemodynamic resuscitation strategy is emphasised to avoid volume overload and not delay the use of norepinephrine in persistent hypotension. Additionally, we recommend addressing haematological and respiratory complications, including platelet transfusion restrictions and strict intra-abdominal pressure monitoring. In more severe cases, plasma exchange and renal replacement therapies are recommended. Based on evidence and the GRADE methodology, implementing these strategies aims to improve survival and reduce morbidity in critically ill yellow fever patients.

## Introduction

The expansion of human activities into jungle areas due to mining, agriculture, and oil extraction, coupled with the impact of climate change, has facilitated the spread of tropical diseases such as yellow fever (YF).^1^, ^2^, ^3^, ^4^ In Colombia, where vaccination coverage is limited in some regions, these factors have led to an increase in disease incidence and mortality, with case fatality rates reaching up to 70% in recent outbreaks.^5^^,^^6^ Between 2024 and 2025, an outbreak in rural areas of Tolima, Colombia, recorded 37 confirmed cases with a preliminary case fatality rate of 40.5%, primarily affecting males aged 13–80 years in villages near the *Bosque de Galilea* Regional Natural Park, with no cases reported in urban areas.^7^ Although historically endemic in South America, with predominant transmission in the Amazon region, its persistence in non-immunised populations highlights the need to strengthen vaccination coverage in at-risk areas.^8^ Now, this has become a public health problem that needs immediate action to provide the best care to these patients.

**Fig. 1:**
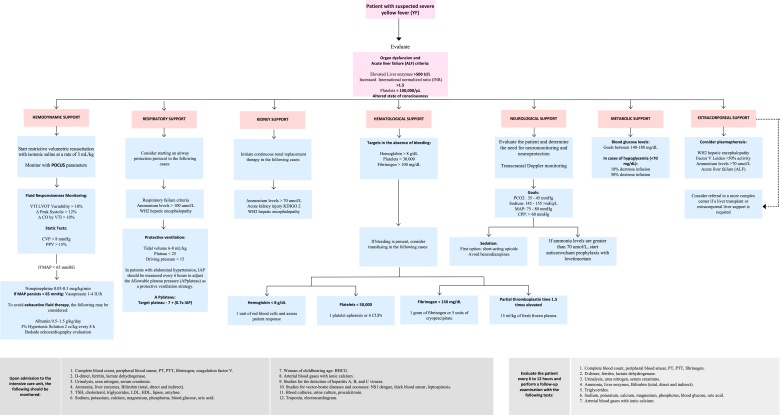
This figure presents an evidence-based clinical algorithm for managing critically ill patients with suspected or confirmed severe yellow fever (SYF) in the intensive care unit. The algorithm stratifies care across seven major domains—haemodynamic, respiratory, renal, haematologic, neurologic, metabolic, and extracorporeal support—each represented by distinct columns. Entry into the algorithm is based on defined criteria for organ dysfunction or acute liver failure (e.g., AST/ALT >500 U/L, INR >1.5, platelets <100,000/μL, altered mental status). Decision points specify clinical thresholds for interventions, such as norepinephrine initiation when MAP <65 mmHg, airway protection for hepatic encephalopathy ≥ grade II, CRRT for ammonia >70 μmol/L, and platelet transfusion thresholds based on bleeding status and procedural needs. This algorithm integrates current best practices, including POCUS-guided fluid management, neuroprotection strategies, and plasma exchange considerations, offering a structured, high-acuity approach to SYF management and follow-up of these patients in resource-limited settings.

Due to the absence of specific antiviral treatment, the management of YF relies on intensive supportive care. Given the lack of targeted therapies and the recent epidemiological alert of YF in the Americas,^9^ standardising clinical approaches are essential. This consensus developed using the GRADE methodology,^10^ provides a framework based on the best available evidence for the diagnosis and management of severe yellow fever (SYF) and is primarily intended for use in highly-complex intensive care unit settings.^11^ Additionally, it incorporates the experience of Hospital Federico Lleras Acosta in Ibagué, the designated reference centre by the Colombian government in Tolima and Colombia, to overcome the public health crisis, aiming to optimise clinical outcomes and improve responses to future outbreaks (Fig. 1).

## Methodology

A structured consensus process integrating the Delphi method^12^ and the GRADE (Grading of Recommendations Assessment, Development and Evaluation) framework was conducted to develop the recommendations for this protocol. A multidisciplinary panel composed of 12 clinical experts (i.e., intensivists, nephrologists, hepatologists, emergency physicians, and epidemiologists) was convened, all with direct experience managing severe YF during the 2024–2025 outbreak in Tolima, Colombia. Each panellist was assigned a specific clinical question, formulated using the PICO structure where applicable. Each expert conducted a focused literature review, including primary studies and systematic reviews relevant to the assigned topic. The quality of evidence was assessed according to GRADE domains, and indirect evidence from analogous conditions (e.g., viral sepsis or acute liver failure) was incorporated with appropriate caution.^13^ Preliminary recommendations were peer-reviewed anonymously by another panellist and modified accordingly. A two-round Delphi process was implemented to refine the recommendations, followed by a face-to-face consensus meeting on February 18, 2025. During the meeting, final recommendations were discussed and submitted to a vote, requiring ≥80% agreement for inclusion. Each recommendation was then rated as strong or weak, in favour or against an intervention, based on the balance of benefits and harms, evidence quality, patient values, and resource considerations. The language was aligned with GRADE guidance: “it is recommended” for strong and “it is suggested” for weak recommendations. This methodology ensured transparency, reproducibility, and contextual relevance in a high-mortality outbreak scenario, allowing for timely and evidence-informed clinical guidance applicable to endemic regions with limited resources.

### Transmission cycle

YF is an RNA arboviral disease caused by the YF Flavivirus. Its transmission occurs through hematophagous arthropod vectors, primarily mosquitoes of the *Aedes aegypti*, *Haemagogus* species and *Sabethes chloropterus*. Three epidemiological transmission cycles are recognised: the sylvatic cycle, where the virus circulates among non-human primates, with the potential for human infection upon contact with vector mosquitoes within forested areas, with *Haemagogus* and *Sabethes* species acting as primary vectors in the Americas.^14^^,^^15^ The intermediate cycle observed mainly in Africa, in which the virus is transmitted to both non-human primates and humans by vectors inhabiting transitional areas. Finally, the urban cycle, where transmission occurs from human to human through infected vectors via *A. aegypti*, a highly anthropophilic mosquito, contributes to large outbreaks in densely populated areas. The latter cycle represents the primary driver of epidemic outbreaks.^16^ Recent evidence highlights the increasing relevance of a peri-urban, or “ecotone” transmission cycle of YF, which occurs in transitional zones between sylvatic and urban environments.^17^

In Colombia, a notable outbreak occurred in rural areas near the Bosque de Galilea Regional Natural Park, with 37 confirmed cases and a case fatality rate of 40.5%.^6^ This population is at high risk of spillover from enzootic transmission involving non-human primates and sylvatic mosquitoes, such as *Haemagogus* and *Sabethes* species, into zones with significant anthropogenic activity. This ecotonal interface increases human exposure, particularly in rural or peri-urban communities, and may serve as a conduit for the virus to enter urban settings, potentially triggering larger outbreaks. Recognising this dynamic is critical for understanding YF epidemiology in South America and implementing effective surveillance and vaccination strategies.^15^^,^^17^

### Clinical presentation

YF is an acute viral haemorrhagic disease characterised by a biphasic clinical presentation. The initial phase, known as the acute phase, usually occurs between 3 and 6 days after infection and presents with nonspecific symptoms, including the sudden onset of fever, chills, loss of appetite, muscle aches, and headache. Patients may also experience photophobia and myalgia. Most individuals recover from this stage within a few days, often with mild symptoms; however, approximately 15–25% of cases progress to the toxic phase.^4^

A suspected case of YF is defined as an individual with a history of residence in, travel to, or epidemiological exposure to regions with known yellow fever virus circulation. Such individuals present with acute fever, operationally defined as a sudden onset of temperature ≥38.5 °C lasting less than seven days, accompanied by myalgia, nausea, vomiting, and abdominal pain.^4^^,^^5^ A subset of patients may progress to a toxic phase characterised by clinical and laboratory evidence of acute liver failure, including jaundice, abnormal liver function tests, acute kidney injury, and hepatic encephalopathy. These severe cases require treatment in an intensive care unit.^4^^,^^5^^,^^11^

## Recommendations

### Haemodynamic support

1. **It is recommended** to implement an early bedside evaluation bundle for fluid resuscitation in patients with SYF based on Point-of-Care Ultrasound (POCUS) strategies.
2. **It is recommended** to avoid fluid overload during fluid resuscitation with isotonic crystalloid solutions in patients with severe YF.
3. **It is recommended** not to delay the use of norepinephrine as the first-line vasopressor in patients with YF and shock.
4. **It is suggested** to use hypertonic solutions (3% sodium chloride) and albumin (0·5–1·5 g/kg/day) as intravascular recruitment strategies, with strict serum sodium and chloride levels monitoring.

Fluid resuscitation is essential in septic shock; however, excessive administration compromises haemodynamic coherence, pulmonary mechanics, myocardial function, and renal perfusion. Endothelial dysfunction induced by cytokines (IL-1, IL-6, TNF-α) increases capillary permeability and interstitial oedema, while activation of the renin-angiotensin-aldosterone system and natriuretic peptides exacerbates venous congestion and organ damage.^18^^,^^19^ A positive fluid balance of >2 L within 48 h increases mortality by up to 50%, and volumes >5 L double the risk of renal injury and the need for renal replacement therapy.^20^^,^^21^ Evidence from trials such as FACTT indicates that strategies to avoid fluid overload are associated with a reduction in the duration of mechanical ventilation and the length of stay in the intensive care unit without negatively impacting 60-day mortality.^20^ Therefore, monitoring haemodynamic parameters utilising ultrasound and dynamic indices is recommended to mitigate the risk of iatrogenic fluid overload.^22^ In patients with septic vasoplegia, a condition characterised by α1-adrenergic resistance and nitric oxide dysregulation, the administration of norepinephrine has demonstrated efficacy in improving perfusion without inducing iatrogenic fluid overload. This effect supports shock reversal and diminishes the likelihood of progression to refractory states.^23^ Furthermore, the findings from the CLOVERS trial and several meta-analyses, including data from over 6500 patients, provide compelling evidence advocating for the early initiation of norepinephrine. These studies have shown associations with reduced mortality rates, a decreased need for mechanical ventilation, and a lower incidence of acute kidney injury. Consequently, it is recommended that norepinephrine be initiated early in the management of persistent hypotension following adequate crystalloid resuscitation to achieve and maintain a mean arterial pressure (MAP) of ≥65 mmHg, thereby mitigating the risk of fluid overload and modulating endothelial inflammation.^24^

Albumin, the primary plasma protein, regulates vascular homeostasis and inflammation. In septic shock, its extravasation leads to relative hypovolemia. Severe hypoalbuminemia (<25 g/dL) is associated with increased respiratory and renal injury, prolonged vasopressor use, and higher mortality rates.^24^ Beyond its oncotic effect, albumin has antioxidant and anti-inflammatory properties, modulating the endothelial glycocalyx and cytokine response.^25^ The ALBIOS trial demonstrated that albumin administration reduces fluid balance and vasopressor dependency, with greater benefit in severe hypoalbuminemia.^26^ A recent meta-analysis showed a 15% reduction in mortality, particularly in acute kidney injury.^27^ In acute liver failure (ALF), the ANSWER study found that prolonged albumin uses decreases mortality and organ dysfunction in decompensated cirrhosis.^28^ The Surviving Sepsis Campaign guidelines, alongside other organisations, recommend albumin administration in septic shock with high crystalloid requirements to achieve haemodynamic stability without fluid overload.^29^

Disruption of the endothelial glycocalyx in SYF contributes to increased capillary permeability, impaired microcirculatory flow, and interstitial oedema. The use of 3% hypertonic saline (HSS) is suggested as an intravascular volume recruitment strategy in patients with SYF and vasoplegic shock, particularly when restrictive fluid management is indicated to avoid positive fluid balance, which has been associated with increased mortality and organ dysfunction.^20^^,^^21^ HSS promotes plasma volume expansion via osmotic gradient-driven fluid shift, optimising preload with lower infused volumes. In addition to its theorical benefits, HSS has been shown to stabilise the endothelial barrier, protect the glycocalyx, and modulate systemic inflammation, thereby mitigating further tissue injury.^30^^,^^31^ In ALF, HSS has effectively reduced intracranial pressure and restored neurovascular homeostasis, counteracting cerebral oedema.^30^ The CRISTAL trial (n = 2857) reported improved 90-day survival with colloid-based strategies despite no significant difference in 28-day mortality,^32^ supporting the rationale for using hypertonic solutions in selected critically ill populations. Garnacho-Montero et al. further endorse HSS as a resuscitation fluid in both septic shock and ALF to achieve haemodynamic stability while minimising interstitial fluid accumulation.^31^ Although there is a lack of robust evidence of yellow fever, the present recommendation is extrapolated from these sepsis and ALF data. Until disease-specific trials are available, the careful, monitored use of 3% HSS may offer physiologic and clinical benefits in this high-mortality context.

### Respiratory support

1. **Early** airway protection via orotracheal intubation is recommended in YF patients with West Haven (WH) encephalopathy >2.
2. Objective measurement of intra-abdominal pressure (IAP) every 6 h is recommended in patients with severe respiratory distress necessitating high ventilatory pressures, a condition frequently associated with fluid overload and suspected intra-abdominal hypertension (IAH). This monitoring is indicated to guide adjustments in permissible plateau pressure within a protective lung ventilation strategy. For this purpose, the following formula is suggested:

Permissible plateau pressure=target plateau pressure−7+(0·7×IAP(mmHg))

Hepatic encephalopathy (HE) is a neurological dysfunction secondary to liver failure that affects cognitive status, consciousness, and motor function. According to the West Haven classification, its stratification guides management. In YF patients with HE grade ≥2, orotracheal intubation should be considered due to the high risk of broncho-aspiration and neurological deterioration.^33^^,^^34^ During fluid resuscitation in YF, fluid administration and capillary leakage promote interstitial oedema, particularly in the intestines, predisposing to IAH and abdominal compartment syndrome, which increases thoracic elastance.^35^ Approximately 50% of IAP is transmitted to the thorax, elevating pleural pressure and increasing resistance to pulmonary expansion. Pelosi et al. propose the formula presented in the recommendations to adjust the permissible plateau pressure in lung-protective ventilation.

### Neurological support

1. Implementing an early neuroprotection bundle based on a multimodal neuromonitoring strategy **is recommended** for the early detection and targeted management of increased intracranial pressure or other neurological complications in SYF patients.
2. **It is suggested** to use anticonvulsant prophylaxis in SYF patients presenting with hepatic encephalopathy of any grade or serum ammonia levels exceeding 70 μmol/L.

SYF is associated with hyperammonaemia, inducing blood-brain barrier disruption and neuronal cytotoxicity, which in severe cases manifests as altered consciousness and intracranial hypertension (ICH). Case series in Brazil have documented hyperacute onset of ALF with rapid progression to encephalopathy and respiratory failure. Given the high lethality and accelerated neurological compromise, early implementation of neuroprotection strategies is crucial. It is recommended to include neuromonitoring and specific measures, as follows:^36^

1. Serial monitoring of serum ammonia levels.
2. Normocapnia (PaCO_2_ 35–40 mmHg).
3. Serum sodium levels maintained between 145 and 155 mEq/L.
4. Head elevation ≥30°.
5. Sedation with short-acting opioids, avoiding benzodiazepines.
6. MAP 75–80 mmHg to ensure a cerebral perfusion pressure (CPP) > 60 mmHg.

Multimodal neuromonitoring is key in neurocritical patients, though few studies have evaluated its outcomes in SYF. Based on ALF guidelines, standardised protocols emphasise non-invasive or minimally invasive strategies.^34^ ICH is a critical complication of ALF secondary to YF. Although ICP monitoring is the diagnostic and therapeutic standard, its use carries a haemorrhagic risk due to coagulopathy. A study of 704 ALF patients (44% due to acetaminophen toxicity) reported intracerebral haemorrhage in 10% following ICP device implantation, with no difference in 30-day post-transplant survival between monitored and non-monitored patients (85% vs. 85%).^34^ European guidelines recommend restricting invasive monitoring to selected cases with grade 3–4 hepatic encephalopathy, refractory ammonia ≥150–200 μmol/L, renal injury, and vasopressor support ≥0·1 μ/kg/min.^34^^,^^37^

Transcranial Doppler ultrasonography is a crucial non-invasive tool in ALF due to its safety in coagulopathy. Validated as an indirect ICP estimator, studies show a strong correlation between pulsatility index (PI) and invasive ICP (r = 0.938; p < 0.0001).^37^ In ALF, PI ≥ 1.0 correlates with an increased risk of severe encephalopathy. Jugular bulb oxygen saturation complements cerebral metabolic assessment in ALF. High oxygen extraction, reflecting conditions like ICH, status epilepticus, and diffuse cerebral oedema, allows early detection and intervention.^38^^,^^39^

The role of anticonvulsant prophylaxis in ALF due to YF remains debated due to a lack of conclusive clinical trials. In a series of 114 patients in Brazil from 2017 to 2018, seizure incidence was 27·2%, but only 9·3% received prophylaxis.^40^ A trial comparing phenytoin vs. placebo in ALF did not significantly reduce seizure incidence (23·8%, p = 0·86) or impact cerebral oedema, mechanical ventilation, or survival.^41^ In 2019, a cohort of 79 SYF patients in Brazil reported an initial seizure incidence of 28%, which decreased to 17% following prophylaxis with levetiracetam and/or lacosamide.^5^ While these findings indicate potential benefits, the absence of controlled studies restricts the ability to generalise these results. It is advisable to utilise non-hepatotoxic anticonvulsants in patients with hepatic encephalopathy and ammonia levels exceeding 70 μmol/L following Brazilian clinical practices.

### Extracorporeal support and liver transplantation and immunologic control

1. **It is recommended** to initiate early renal replacement therapy (RRT) in patients with grade 2 hepatic encephalopathy (West Haven scale), serum ammonia levels above 70 μmol/L, or severe acute kidney injury.
2. **Continuous renal replacement therapies** (CRRT) over intermittent therapies are recommended.
3. **It is recommended** to use plasma exchange in patients with YF and ALF or serum ammonia levels above 70 μmol/L as a bridge-to-recovery strategy.
4. **No recommendation** is issued for or against using cytokine removal therapies in SYF patients.
5. **No recommendation** is issued for or against implementing extracorporeal liver support strategies (SPAD, MARS, Prometheus) in SYF patients.
6. **No recommendation** is issued for or against referral for liver transplantation in patients with ALF due to SYF.
7. **It is suggested** to initiate high-dose corticosteroid therapy with methylprednisolone or equivalent in patients with SYF and a positive HScore (>160) as part of an immunomodulatory strategy for secondary hemophagocytic lymphohistiocytosis (sHLH), followed by gradual tapering.

In YF patients, neurological manifestations during ALF, as classified by West Haven, are associated with hyperammonaemia,^33^ which correlates with cerebral oedema, increased ICP, and higher mortality.^34^^,^^37^^,^^42^^,^^43^ In a multicentre cohort, Cardoso et al. found that elevated ammonia levels correlate with more severe encephalopathy, brain death, and increased overall mortality. During the first three days of CRRT, a significant reduction in ammonia levels, lower mortality, and a higher probability of avoiding liver transplantation at 21 days were observed, an effect not seen with intermittent therapies.^42^ Slack et al. reported that ammonia reduction with CRRT correlated with ultrafiltration rate (R = 0·86).^44^ SYF is a rapidly progressing disease that can lead to multiple organ dysfunction. Current recommendations emphasise early interventions to correct critical complications such as coagulopathy and hepatic encephalopathy.^5^

Plasma exchange has emerged as an alternative to modulate coagulopathy and reduce serum bilirubin and ammonia levels. The European Association for the Study of the Liver (EASL) (2017) recommends its use to improve survival and prolong transplant-free days, supported by Larsen et al. (2016), where high-volume plasma exchange in ALF due to acetaminophen toxicity showed benefits in patients with encephalopathy grade ≥2.^45^ Ho et al. evaluated intensive plasma exchange vs. high-volume plasma exchange and conventional management, demonstrating an 84% reduction in mortality.^46^ Although high-volume plasma exchange has been the standard, recent data suggest that a daily strategy could optimise outcomes in YF.^46^

Extracorporeal liver support therapies (MARS, SPAD, and Prometheus) lack sufficient evidence to reduce mortality or improve clinical outcomes in YF. Previous studies in ALF of other aetiologies have shown inconsistent results, failing to demonstrate benefits in reversing multiple organ dysfunction or improving survival.^47^^,^^48^ While the therapies above are primarily used to remove toxins and support hepatic detoxification, their effects are limited to these functions. In contrast, YF involves not only liver failure but also a severe cytokine storm along with a profound coagulopathy, leading to further endothelial injury, haemorrhagic complications, and multi-organ failure.^49^^,^^50^ Currently, none of the available devices fully address these complex pathophysiological processes.^51^^,^^52^

Liver transplantation is a highly complex procedure with high costs and significant perioperative risks. During the YF epidemic in Brazil (2016–2018), liver transplants were performed in 30 patients with ALF secondary to infection, with a survival rate of 20% (6 patients).^36^^,^^53^ Given the high resource burden, the need for transplant-experienced centres, and advanced postoperative intensive care, it is not currently considered a viable therapeutic strategy at the population level or a recommended option in SYF management guidelines. However, isolated cases have been reported in highly selected cases and institutions capable of optimising immunosuppression and multi-organ support. Further studies are needed to define its impact on specific populations and its potential role in the treatment algorithm.

In patients with SYF presenting with hyperinflammatory features and a positive HScore suggestive of secondary hemophagocytic lymphohistiocytosis (sHLH), it is suggested to initiate high-dose corticosteroid therapy as an immunomodulatory intervention. Methylprednisolone is recommended as the first-line agent due to its capacity to inhibit dendritic cell maturation, downregulate pro-inflammatory cytokines (e.g., IL-1β, IL-6, TNF-α, IFN-γ), and suppress COX-2 and prostaglandin E2 pathways implicated in viral immune pathology.^54^, ^55^, ^56^ Upon achieving initial control, gradual tapering is advised to reduce rebound inflammation. This approach is supported by pathophysiological similarities between HLH and flavivirus-induced hypercytokinemia and by clinical analogies drawn from dengue and vaccine-related viscerotropic disease, where corticosteroid use has shown potential survival benefit.^56^, ^57^, ^58^ Given the high risk of rapid multiorgan failure, early recognition and prompt corticosteroid initiation may attenuate immunopathology and improve outcomes in this subgroup.

### Haematologic support

1. **We recommended against** the routine or prophylactic use of fresh frozen plasma, other clotting factors, or platelets to correct conventional coagulation tests.
2. Fresh frozen plasma or other coagulation factors **are recommended** in cases of active bleeding or medical intervention requirements.
3. Maintaining a platelet transfusion threshold of 30,000 platelets/μL in SYF patients with organ dysfunction **is recommended**. In cases of active bleeding or patients receiving extracorporeal support therapies, increasing the transfusion threshold to 50,000 platelets/μL is recommended.
4. **It is recommended** that haemoglobin levels ≥8 g/dL be maintained in critically ill patients with organ dysfunction, prioritising a restrictive transfusion strategy in the absence of shock or persistent hypoperfusion.
5. Maintaining fibrinogen levels ≥100 mg/dL in critically ill YF patients without bleeding and levels ≥150 mg/dL in those undergoing extracorporeal therapies is **recommended**.

The mechanisms of coagulopathy in YF are multifactorial and distinct from those observed in other causes of fulminant hepatitis or viral haemorrhagic fevers such as dengue. Meanwhile, thrombocytopenia, disseminated intravascular coagulation (DIC), and hepatic dysfunction contribute to bleeding in YF.^59^ In a study by Franco et al., among 46 patients with yellow fever, 35% of those with severe disease died. Bleeding occurred in 45% of cases, with severe haemorrhage in 32%. Mortality was associated with prolonged INR and aPTT and significantly decreased levels of coagulation factors II, V, VII, IX, and protein C (p < 0.05). D-dimer levels were ten times higher in severe patients than in moderate disease (p < 0.01). The authors concluded that severe YF is characterised by a deficiency of liver-derived coagulation factors, contributing to the observed bleeding diathesis.^60^

ALF induces multifactorial thrombocytopenia through a cascade of events mediated by inflammatory endothelial dysfunction, leading to coagulation activation and platelet consumption. This process is exacerbated by hemophagocytosis, decreased platelet production due to hepatic dysfunction, splenic sequestration, and increased platelet aggregation from ADAMTS13 deficiency. Additionally, platelet destruction mediated by autoantibodies contributes to disease severity.^61^ In patients with yellow fever, elevated YFV NS1 and syndecan-1 serum levels correlated with disease severity, endothelial dysfunction, viral load, and mortality. NS1-induced vascular hyperpermeability and syndecan-1 shedding in endothelial cells. These findings support NS1 and syndecan-1 as potential prognostic biomarkers and contributors to yellow fever pathogenesis.^62^

In YF patients, a restrictive transfusion strategy is recommended, guided by bleeding risk and haemodynamic stability. An analysis of the HALT-C trial, which included 2740 liver biopsies, reported minimal bleeding (0·6%) in patients with platelet counts >50,000/μL. In the context of extracorporeal therapies, this threshold is appropriate, whereas, in critically ill patients with severe thrombocytopenia, transfusion is indicated for counts <30,000/μL or <50,000/μL in cases of active bleeding.^63^

In viral sepsis, immune activation initiates a pro-inflammatory response that shortens erythrocyte lifespan and suppresses erythropoiesis through immune-mediated mechanisms and endothelial dysfunction. Systemic inflammation impairs red blood cell synthesis by disrupting iron metabolism, resulting in progressive anaemia. Regarding haemoglobin maintenance, sustaining a threshold of >8 g/dL in critically ill YF patients is recommended, extrapolating evidence from other critical care settings where this level was associated with reduced mortality without increasing complications. In YF, maintaining this level optimises tissue oxygenation, mitigates multi-organ dysfunction, and reduces mortality associated with ALF.^5^^,^^64^

YF induces severe coagulopathy mediated by the overexpression of pro-inflammatory cytokines such as TNF-α and IFN-α, whose concentrations correlate with endothelial damage severity, thrombocytopenia, and disseminated intravascular coagulation. TNF-α activates the extrinsic coagulation pathway by inducing tissue factor expression, generating a paradoxical prothrombotic state that is simultaneously associated with higher bleeding risk. IFN-α, in addition to modulating the immune response, compromises the hepatic synthesis of coagulation factors in SYF, further aggravating the haemostatic imbalance.^15^^,^^59^^,^^65^

Fibrinogen, a key glycoprotein in haemostasis, is synthesised in the liver and regulates both coagulation and inflammation. Its maturation involves post-translational modifications that determine its functionality and platelet interactions. In YF, severe hepatic damage results in hypofibrinogenemia and dysfibrinogenemia, characterised by aberrant hypersalivation that alters its conformation and functionality, promoting unstable thrombus formation and haemorrhagic events. Additionally, fibrinogen modulates innate immunity and fibrinolysis, playing a central role in coagulopathy progression. Severe hypofibrinogenemia (<100 mg/dL) increases bleeding risk and requires correction in critical scenarios to mitigate haemorrhagic complications.^61^

### Antiviral therapy

1. **No recommendation is made for or against** implementing targeted antiviral strategies in SYF patients. To date, no clinical trial has demonstrated the efficacy of any antiviral drug for preventing or treating YF-related outcomes.

Currently, YF treatment is based on supportive measures, as no antiviral has been approved with proven efficacy in humans. However, recent research has explored the use of nucleotide analogues such as sofosbuvir, remdesivir, and uprifosbuvir, which have shown in vitro inhibitory activity against the virus but lack validation in controlled clinical trials.^66^^,^^67^ Similarly, galidesivir has demonstrated uniform inhibition in human cell lines and protection in animal models infected with flaviviruses, but its clinical efficacy in humans remains unestablished.^68^ The available evidence does not support the recommendation of targeted antiviral strategies in SYF.^69^ Robust clinical trials are needed to evaluate their impact on disease progression. Meanwhile, vaccination remains the primary preventive strategy, and intensive care management is crucial for patients with severe forms of the disease.

## Conclusion

This consensus underscores the critical nature of severe yellow fever, which is characterised by high mortality and rapid multisystem deterioration. It necessitates an interdisciplinary and individualised approach supported by the highest diagnostic and therapeutic level of care in intensive care units. The consensus emphasises the importance of early, goal-directed intervention strategies informed by ultrasound within a multimodal monitoring protocol.

Extracorporeal therapies, such as plasma exchange and continuous renal replacement therapy, have demonstrated the most compelling evidence for supporting liver dysfunction and managing severe hyperammonaemia. Immunological control through steroid use also remains a cornerstone in treating SYF; however, further trials are needed to clarify this approach. Given this condition's progressive and severe nature, a rapid multidisciplinary treatment is essential to mitigate complications and improve clinical outcomes. This consensus outlines an optimised approach based on the best available evidence while reinforcing the need for ongoing research to improve treatment strategies and strengthen preventive measures against Yellow Fever.^70^

## Contributors

**Conceptualisation:** AJFD, JAMO, JFCG, OEZD, GAGV, CAAB, RSF, JOO, HR-A, AQA, DCRR, JDM-P, JMF, JOG, AKZ, WEVG, AJR-M, RBB, FB, LFR. **Data curation:** AJFD, JAMO, JFCG, OEZD, GAGV, CAAB, RSF, JOO, HR-A, AQA, DCRR, JDM-P, JMF. **Formal analysis:** AJFD, JAMO, JFCG, OEZD, GAGV, CAAB, RSF, JOO, HR-A, AQA, DCRR, JDM-P, JMF, JOG, LFR. **Funding acquisition:** JOG, LFR. **Investigation:** AJFD, JAMO, JFCG, OEZD, GAGV, CAAB, RSF, JOO, HR-A, AQA, DCRR, JDM-P, JMF, LFR. **Project administration:** LFR. **Software**: AJFD, JAMO, JFCG, OEZD, GAGV, CAAB, RSF, JOO, LFR. **Supervision:** LFR. **Validation:** AJFD, JAMO, JFCG, OEZD, GAGV, CAAB, RSF, JOO, LFR. **Visualization:** AJFD, JAMO, JFCG, OEZD, GAGV, CAAB, RSF, JOO. **Writing – original draft:** AJFD, JAMO, JFCG, OEZD, GAGV, CAAB, RSF, JOO, JOG. **Writing – review & editing:** AJFD, JAMO, JFCG, OEZD, GAGV, CAAB, RSF, JOO, JOG, AKZ, WEVG, AJR-M, RBB, FB, LFR.

All authors have approved the submitted version and agreed to be personally accountable for the author's contributions and to ensure that questions related to the accuracy or integrity of any part of the work.

## Declaration of interests

Authors declare no competing interests.
